# The Influence of DNA Extraction Methods on Species Identification Results of Seafood Products

**DOI:** 10.3390/foods11121739

**Published:** 2022-06-14

**Authors:** Rosalía Rodríguez-Riveiro, Amaya Velasco, Carmen G. Sotelo

**Affiliations:** Instituto de Investigaciones Marinas (CSIC), Eduardo Cabello 6, 36208 Vigo, Spain; amayavelasco@iim.csic.es (A.V.); carmen@iim.csic.es (C.G.S.)

**Keywords:** DNA extraction methods, Cephalopoda, Gadiformes, Pleuronectiformes, polymerase chain reaction (PCR), sequencing

## Abstract

In terms of species identification, the ultimate aim of extracting DNA is the subsequent amplification of the selected marker; therefore, the quality and quantity of the extracted DNA must be sufficient for PCR-based methods. The purpose of this study is to compare five DNA extraction methods according to the parameters of quantity, quality and simplicity, among others, in order to determine the most suitable method for identification for Cephalopoda, Gadiformes and Pleuronectiformes. The Wizard DNA clean-up system kit (Promega), MPure-12^TM^ automated nucleic acid purification system (MP Biomedicals), Chelex 100 resin (Biorad), DNeasy blood and tissue kit (Qiagen) and a swab method were examined. The obtained DNA quantity was determined by fluorescence, and quality was evaluated with ratios of absorbance of A260/A280 and A260/A230 by agarose gel visualization of the extracts and by analyzing the success of PCR amplifications of 720 bp fragments of cytochrome c oxidase I (COI) for Cephalopods and 465 bp fragments of cytochrome b for Gadiformes and Pleuronectiformes. Statistical results confirmed significant differences between the tested methods according to yield, efficiency and purity and no significant differences with respect to the species employed. The best yields were obtained with the Wizard kit, whereas other methods stand out in terms of their affordability (Chelex) and automation (Mpure).

## 1. Introduction

DNA extraction is of paramount importance in the workflow of any DNA-based methodology [1,2,3]. Various DNA-based methodologies are applied to a wide variety of problems, and DNA can be obtained from numerous different types of tissues and samples [4,5]. DNA sequence analysis is the gold standard for species authentication and it is often preferred over protein analysis because much more information can be obtained from DNA compared with traditional protein analysis [6].

In terms of species identification, the ultimate aim of isolating DNA is the subsequent amplification of the gene, which implies that the quality of the extracts, in addition to the quantity, must be evaluated [7].

Four steps are indispensable in nucleic acid purification: tissue disruption, nucleoprotein denaturation, nuclease inactivation and removal of contaminants and polymerase chain reaction (PCR) inhibitors [8]. Once extracted, the isolated DNA can be used in numerous PCR-based applications [4]. In spite of the ability of PCR tests to work on poor-quality DNA samples, the presence of other molecules, such as inhibitors, in the DNA extracts can interfere with the results of the amplifications [2,9]. Thus, the DNA must be isolated, purified and concentrated by methods that ensure the removal of the inhibitor in the PCR test [2]. The lower the presence of RNA, PCR inhibitors and DNA fragmentation resulting from the extraction, the more successful the amplification and the method [1]. In addition, the toxicity, the time required and the price of used material are points that must be considered when choosing an isolation technique [7]. Additionally, the choice of extraction methodology is of considerable importance to avoid time-consuming optimization in downstream analysis. Moreover, it is necessary to take into account the quantity and quality of DNA required according to the application [1,4]. Some factors to consider when choosing an appropriate method include sample characteristics (amount, type, processing degree and origin), cost, length, simplicity of the process (this affects the DNA integrity), equipment required [1,4], toxicity and disposal of reagents, DNA yield and purity [1].

DNA analysis has become widely used in recent years for the identification of seafood [10]. Because the morphology of some species is transformed during the processing of commercial products, the visual assignment of species in the case of some food products becomes extremely difficult. In addition to the high commercial value of some marine species, this leads to frequent seafood mislabeling [6,11]. Therefore, an accurate authentication method is crucial to avoid fraud in the fishing sector [12,13]. Some comparisons of DNA extraction methods have been carried out for general samples and for food matrices [2,4], but so far, no specific study has been conducted for seafood.

Cephalopoda, Gadiformes and Pleuronectiformes are relevant groups in the world seafood trade. Cephalopods are one of the most important groups in terms of catches, with 322,000 tons in 2018 [14]. In recent years, the world catches of the common cuttlefish (*Sepia officinalis*) ranged between 8500 and 14,000 tons, reaching 24,059 tons in 2019. This species, which is mainly commercialized frozen or fresh, is very appreciated in some countries, such as Japan and Spain [15]. As an example of Gadiformes, *Gadus morhua* is one of the most important commercial fishes in the world. It is marketed under a wide variety of presentations, and in 2018, catches totaling 1,218,000 tons were reported [14]. *Merluccius merluccius* is also relevant in this group and can be found fresh, frozen, salted, dried and canned in markets of western Europe, reaching a global production of 116,889 tons in 2019. France, Portugal and Spain are the main producers and consumers of *Scophthalmus maximus*. Spain, the top global producer, accounted for 75.9% of the global production (3847 tons) in 2002 [15]. However, despite the enforcement of seafood labeling regulations, species substitution remains common [11]; therefore, it is important to develop fast and reliable methods of effective seafood authentication.

The DNA molecule has proven to be stable at different temperatures, owing to its ability to reveal the identity of food species present in different processed foodstuffs [10]. DNA extraction methods have evolved over the years, so there is now a diverse variety of commercial kits available with different characteristics and targeting different types of samples. The aim of this study is to compare DNA extraction methods in order to determine the most suitable method for identification of relevant groups of seafood species (Cephalopoda, Gadiformes and Pleuronectiformes). This objective is essential because DNA extraction is often the first step in seafood authentication and traceability control methods. To that end, different parameters, such as DNA yield and quality, automation, simplicity and suitability for subsequent amplification, were evaluated.

## 2. Materials and Methods

### 2.1. Sampling

Specimens of marine invertebrates and vertebrates of the groups Cephalopoda *(Sepia officinalis*), Gadiformes (*Merluccius merluccius*, *Gadus morhua*) and Pleuronectiformes (*Scophthalmus maximus*) were purchased fresh at a local fish market in Vigo, Spain. Three specimens per species were obtained, for a total of 12 samples used in this study. Specimens were visually identified and photographed before further processing.

### 2.2. Sample Processing

Samples of the edible portion of the specimens (without skin or bones) were cut into small sections, homogenized using a food processor (Thermomix) and distributed in plastic bags, which were stored at −80 °C for at least 24 h before DNA isolation in order to ensure the same starting material for the different extraction methods. Homogenized samples were thawed at room temperature before DNA extraction. Because the focus of our study is verifying the authenticity of seafood products, muscle tissue samples were used, as they represent the edible part of most fishery products.

### 2.3. DNA Extraction

The following methodologies based on different principles were used for DNA extraction of all samples. These methods were selected because for their rapidity, safety, affordability and automation, as well as their efficiency in extracting DNA from muscle tissue of the investigated groups of organisms.

#### 2.3.1. Wizard DNA Clean-Up System Kit (Promega, Madison, WI, USA)

This methodology involves the use of a resin with DNA binding capacity and subsequent purification. A portion of 0.3 g of tissue was cut, minced with a scalpel and placed in a 1.5 mL Eppendorf tube with 860 µL of extraction buffer (1% sodium dodecyl sulfate (SDS), 150 mM NaCl, 2 mM ethylenediaminetetraacetic acid (EDTA) and 10 mM Tris-HCl at pH 8), 100 μl 5M guanidinium thiocyanate and 40 µL proteinase K (20 mg/mL). After vortexing, samples were incubated for 2 h in a thermomixer at 56 °C and 800 rpm [6]. Then, the protocol indicated by the manufacturer was followed for DNA isolation. For this method and the others, the conditions of incubation were selected according to the manufacturer’s recommendations and also derived from previous experiments employing this type of method and organisms [7]. These conditions proved to be the most suitable for proper DNA extraction.

#### 2.3.2. MPure-12^TM^ Automated Nucleic Acid Purification System (MP Biomedicals, Santa Ana, CA, USA)

An MPure tissue DNA extraction kit (MP Biomedicals, Santa Ana, CA, USA) was used with an MPure-12^TM^ automated nucleic acid purification system. The principle of the method involves magnetic bead separation technology. Following the protocol indicated by the manufacturer, 40 mg of tissue were cut with a scalpel and placed in a 1.5 mL Eppendorf tube with 400 µL of BL2 buffer and 20 µL proteinase K (20 mg/mL). After vortexing, samples were incubated for 2 h in a thermomixer at 56 °C and 1000 rpm. After digestion, 400 μL of digested tissue was introduced into the sample tube, and DNA was extracted automatically by the MPure-12 instrument.

#### 2.3.3. Chelex 100 resin (Biorad, Hercules, CA, USA)

Chelex resin traps metal ions and other possible contaminants present in the sample, leaving a DNA in solution. Following the protocol indicated by the manufacturer with some modifications according described by Sepp [9], approximately 10 mg of sample was vortexed in 100 µL of a 5% Chelex solution. Samples were centrifuged at 14,000 rpm for 2 min and incubated at 95 °C for 20 min. Then, tubes were vortexed and chilled on ice. Finally, DNA suspension was centrifuged at 14,000 rpm for 2 min and stored at 4 °C.

#### 2.3.4. DNeasy Blood and Tissue Kit (Qiagen, Hilden, Germany)

The principle of this method is a silica-based extraction in spin columns. Following the protocol indicated by the manufacturer, tissue samples of 25 mg were weighed into a microcentrifuge tube and vortexed with 180 μL ATL buffer and 20 μL proteinase K (20 mg/mL). Then, samples were incubated at 56 °C for 2 h, and 200 μL of AL buffer and 200 μL of ethanol (96–100%) were added. Samples were then transferred to silica columns, centrifuged and transferred to new collection tubes. Aliquots of 500 μL of AW1 wash buffer and AW2 wash buffer were added sequentially and centrifuged. Silica columns were transferred to microcentrifuge tubes, and 200 μL of AE buffer was added to each column. Columns were incubated at room temperature for 1 min and centrifuged DNA elution.

#### 2.3.5. Swab Method

This procedure involved no tissue digestion or DNA isolation. A sterile cotton swab was placed into an Eppendorf tube and weighed. Then, the swab was dipped into the homogenized sample and placed in the Eppendorf tube to be weighed again to determine the amount of sample absorbed into the swab. Then, 500 µL of water was added to the tube, and the swab was manually stirred for 30 s. The stick was then cut off, and only the absorbing part of the swab was left inside the tube. The sample was centrifuged at 14,000 rpm for 5 min without removing the swab, and the supernatant was used for the subsequent PCR test without further isolation, resulting in an extract that was ready to use.

### 2.4. DNA Quantity and Quality Determination

#### 2.4.1. Yield and Efficiency

The extracted double-stranded DNA was quantified with and Invitrogen Qubit 4 fluorometer (ThermoFisher Scientific, Waltham, MA, USA) using a Qubit dsDNA BR assay kit (Invitrogen, Waltham, MA, USA). DNA yield was calculated by multiplying the DNA concentration value by the final volume of DNA extracted by each method. Moreover, due to the differences in the initial amount of tissue among methods according to the recommendations of manufacturers and previous studies [7], the method efficiency was determined by dividing the DNA yield by the tissue weight (wet basis).

DNA yield (ng) = [DNA] (ng/µL) × DNA extracted (µL)

Method efficiency (ng/mg) = DNA yield (ng)/tissue weight (mg)

#### 2.4.2. Purity

Purity was determined with a Nanodrop 2000 spectrophotometer (ThermoFisher Scientific, Waltham, MA, USA) with ratios of absorbance of A260/A280 and A260/A230 [2]. The ranges considered optimal were 1.8–2.0 for the ratio A260/A280 and 1.8–2.2 for the ratio A260/A230 [16].

#### 2.4.3. Integrity of Extracted DNA

Extract quality in terms of DNA fragmentation was determined by running extracts through a 1% (*w*/*v*) agarose gel. For each sample, 10 microliters was loaded [200 ng]. This concentration was selected on the basis of a test to determine the minimum concentration of DNA extract that can be visualized in the gel. The size of the DNA was estimated according to the GeneRuler 100 bp DNA ladder standards (ThermoFisher Scientific, Waltham, MA, USA) and the Lambda DNA/HindIII marker (ThermoFisher Scientific, Waltham, MA, USA).

### 2.5. Handling Time and Total Extraction Time

The total extraction time required for each DNA extraction methodology was calculated from the sum of the digestion time and the handling time. In this study, the digestion time was always 2 h, but the handling time depended on the protocol. Because of technical specifications of the Wizard and Mpure-12 protocols, these methods were applied simultaneously to 10 and 12 samples, respectively, with the protocols optimized for these quantities; therefore, the handling time for one sample was similar to the time spent for 10 or 12 samples individually.

### 2.6. PCR Amplification and Sequencing

#### 2.6.1. PCR

To test the suitability of the extracted DNA for amplification, polymerase chain reactions were carried out in a Veriti thermal cycler (Applied Biosystems, Waltham, MA, USA). Because it is an universal technique for the identification of all species and the routine method used in most control laboratories, PCR was the chosen technique [17,18]. The importance of including very genetically distant groups of species, such as cephalopods and fish, implied the selection of different pairs of primers for each group. For cephalopods, Folmer primers [19] were chosen to amplify a 720-base-pair (bp) fragment of cytochrome c oxidase I (COI) (LCO1490-5′ GGTCAACAAATCATAAAGATATTGG3′ and HCO2198-5′ TAAACTTCAGGGTGACCAAAAAATCA3′). PCR was conducted under the following thermal cycling conditions: a preheating step of 94 °C for 5 min, followed by 35 cycles of 94 °C for 40 s, 48 °C for 1 min 20 s, 72 °C for 1 min 20 s and a final extension at 72 °C for 7 min. For Gadiformes and Pleuronectiformes groups, Burgonet primers [20] were used to amplify a 465 bp fragment of cytochrome b (L14735-5′AAAAACCACCGTTGTTATTCAACTA3′ and H15149ad-5′GCICCTCARAATGAYATTTGTCCTCA3′). In this case, the thermal conditions were as follows: initial denaturation at 94 °C for 5 min, followed by 35 cycles of denaturation at 94 °C for 40 s, primer annealing at 55 °C for 1 min 20 s and chain elongation at 72 °C for 1 min 20 s, with a final extension at 72 °C for 7 min. PCR reactions (final volume, 25 µL) were prepared with Illustra PuReTaq Ready-To-Go PCR beads (GE Healthcare, Chicago, IL, USA), with 1 µL (10 µM) of each primer and 2–5 µL (50 ng/µL) of template DNA, depending on the concentration of DNA obtained with the extraction method used. In addition, a negative PCR control without template DNA was included in all tests. For the determination of the amplification success, PCR products were visualized in a 2% (*w*/*v*) agarose gel with a 5 µL loading of each PCR product. The size of the amplified fragments was estimated from the molecular marker GeneRuler 100 bp DNA ladder (ThermoFisher Scientific, Waltham, MA, USA).

#### 2.6.2. Sanger Sequencing

Positive PCR reactions were purified with an Illustra ExoProStar one-step kit (GE Healthcare, Chicago, IL, USA) following the manufacturer’s protocol. After PCR purification, both strands were sequenced in an ABI 3730 xl automatic sequencer (Applied Biosystems, Waltham, MA, USA).

#### 2.6.3. Sequence Quality Determination

For the study of sequence quality, sequences were trimmed with the Geneious program (Bioinformatics Software for Sequence Data Analysis) to ensure a consistent consensus read length (372 bp for Cytochrome b and 619 bp for COI) in order to obtain an estimation of the average quality for each sequence.

#### 2.6.4. Species Authentication

The obtained sequences were also used for authentication of individuals by FINS (forensically informative nucleotide sequencing). Phylogenetic trees were created using the neighbor-joining method with the Tamura–Nei model with 1000 bootstrap replicates [21].The sequences were also checked with the BLAST tool of the NCBI (National Center for Biotechnology Information) to confirm the species.

### 2.7. Complementary Parameters

#### 2.7.1. Safety

The safety of the DNA extraction kits was estimated based on the specifications of each method. Hazardous components and substance classifications of each method were checked for comparison.

#### 2.7.2. Affordability

The affordability the methodologies varied depending on the specific materials and equipment required to carry them out. The cost per sample was calculated, and the value of the materials associated with each extraction method was studied for later comparison.

#### 2.7.3. Simplicity

The working complexity of each DNA extraction method was evaluated from a technical point of view. 

#### 2.7.4. Automation

The possibility of automating the methodologies was investigated—an important parameter to consider when working with a large number of samples.

### 2.8. Statistical Analyses

Yield, efficiency and purity data were statistically analyzed using the SPSS Statistical Software System 28.0.1.0 (142). First, the Kolmogorov–Smirnov test was carried out to test the normality of the data distribution. A logarithmic function was used to transform the data that did not follow a normal distribution. To compare significant differences of yield, efficiency and purity obtained with the different extraction methods, a one-way analysis of variance (ANOVA) was used. The same statistical analysis was performed to compare data among species. In both cases, when significant differences were observed, the Bonferroni correction for multiple comparisons was used to determine the effect of the interactions. In addition, to study the method–species interaction, a two-way ANOVA was carried out, and the Tukey test was used to verify the interactions of the values with significant differences. Differences were considered statistically significant at the 5% level (*p* < 0.05). Replicates were considered as individual samples in all the statistical analyses.

## 3. Results

### 3.1. DNA Quantity and Quality

#### 3.1.1. Yield and Efficiency

**Figure 1:**
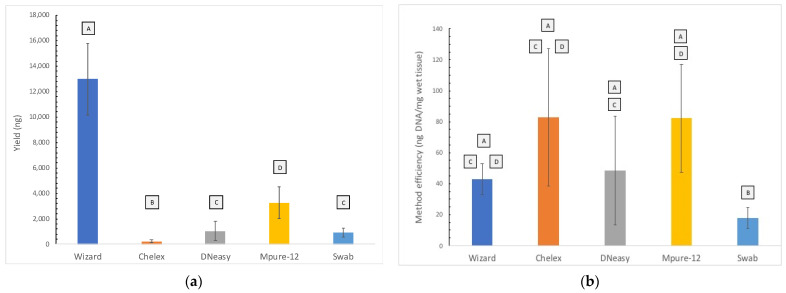
(**a**) Yield (ng) of the tested DNA extraction methods. Data are presented as mean ± SD of samples per method. Non-identical subscript letters (A, B, C, D) indicate a statistically significant difference (*p* < 0.05). (**b**) Method efficiency (ng DNA/mg wet tissue) of the tested DNA extraction methods. Data are presented as mean ± SD of samples per method. Non-identical subscript letters (A, B, C, D) indicate a statistically significant difference (*p* < 0.05).

The different DNA extraction methods were compared in terms of total DNA extracted (yield) and efficiency. Figure 1a presents the yield for each method studied. The highest yield was obtained with the Wizard method (12,979 ± 2805 ng). Despite the low yield obtained with the Chelex method, it was the method with the highest efficiency (83 ± 44 ng DNA/mg wet tissue), closely followed by MPure-12 (82 ± 35 ng DNA/mg wet tissue). The efficiency of these two methods differs significantly from that obtained with the swab methodology (Figure 1b).

**Figure 2:**
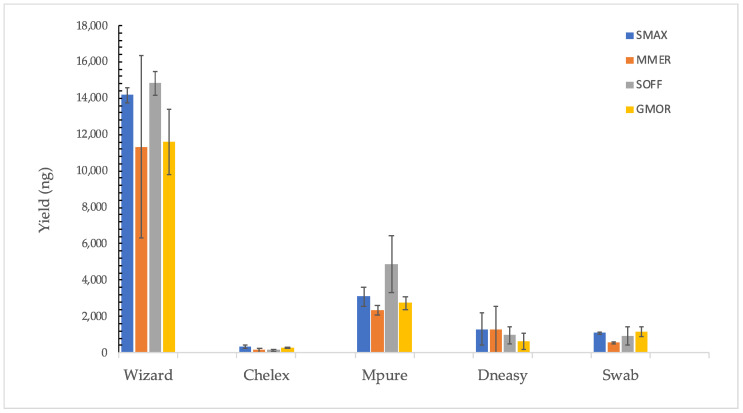
Yield (ng) of the tested methods per species. Data are presented as mean ± SD of individuals per species. *Scophthalmus maximus* (SMAX), *Merluccius merluccius* (MMER), *Sepia officinalis* (SOFF) and *Gadus morhua* (GMOR).

**Figure 3:**
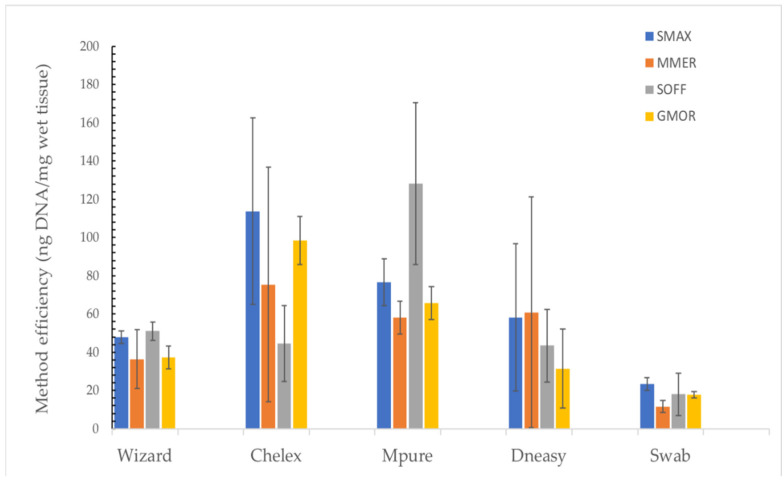
Method efficiency (ng DNA/mg wet tissue) of the tested methods per species. Data are presented as mean ± SD of individuals per species. *Scophthalmus maximus* (SMAX), *Merluccius merluccius* (MMER), *Sepia officinalis* (SOFF) and *Gadus morhua* (GMOR).

The Wizard method was found to be the most suitable protocol to achieve high yields for all of the tested species, with the best yield value of all samples obtained for *Sepia officinalis* (14,817 ± 625) by Wizard extraction (Figure 2). Among all tested methods, Chelex was, on average, the extraction method with the best efficiency (Figure 3). However, the MPure-12 method showed similar results and worked better for *Sepia officinalis* (128 ± 42 ng/mg).

#### 3.1.2. Purity

**Figure 4:**
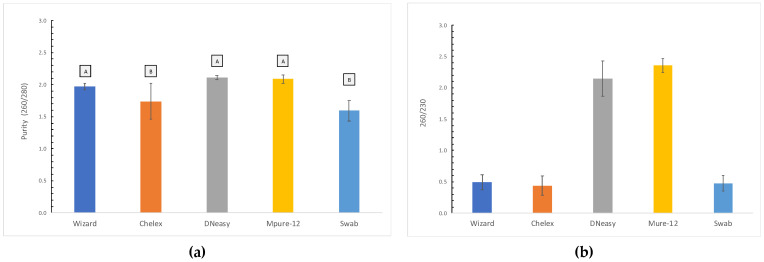
(**a**) Purity (260/280) of the tested DNA extraction methods. Data are presented as mean ± SD of samples per method. Non-identical subscript letters (A, B) indicate a statistically significant difference (*p* < 0.05). (**b**) Ratio 260/230 of the tested DNA extraction methods. Data are presented as mean ± SD of samples per method.

The purity of the extracted DNA was verified by measuring its absorbance at 230, 260 and 280 and evaluating the ratios 260/280 and 260/230. In the case of the ratio 260/280, the method with the closest values to the optimal 1.8–2 range was Wizard (Figure 4a), although no significant differences were observed between the Wizard, DNeasy and MPure-12 methods. However, in the case of the 260/230 ratio, only the results from DNeasy and MPure-12 are close to the optimal range (1.8–2.2) (Figure 4b).

**Figure 5:**
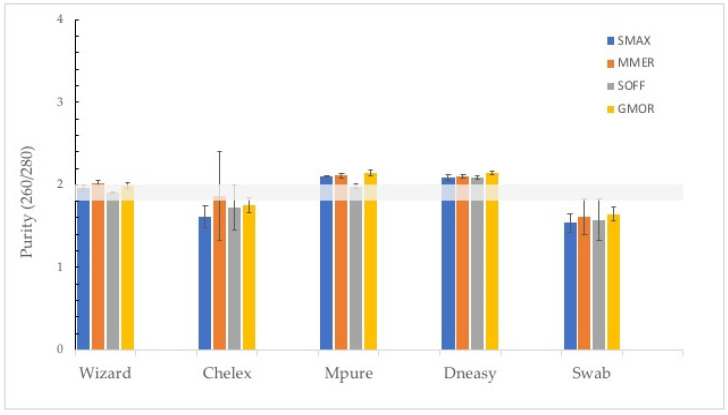
Purity (260/280) of the tested DNA extraction methods per species. Data are presented as mean ± SD of individuals per species. *Scophthalmus maximus* (SMAX), *Merluccius merluccius* (MMER), *Sepia officinalis* (SOFF) and *Gadus morhua* (GMOR).

**Figure 6:**
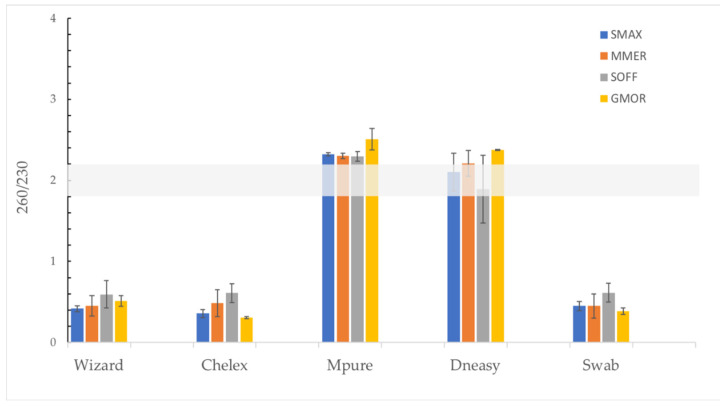
Ratio 260/230 of the tested DNA extraction methods per species. Data are presented as mean ± SD of individuals per species. *Scophthalmus maximus* (SMAX), *Merluccius merluccius* (MMER), *Sepia officinalis* (SOFF) and *Gadus morhua* (GMOR).

The analysis per species of the purity of the DNA extracts is presented in Figure 5. The extraction protocol that performed the best for all species was the Wizard method (260/280). However, for *Merluccius merluccius* and *Sepia officinalis*, the Chelex and MPure-12 methods, respectively, generated DNA within the optimal purity range (260/280). Most of the DNA extractions of the tested species showed values that indicate the presence of contaminants, regardless of the method used (260/230) (Figure 6). The values of the A260/A230 ratio (1.8–2.2) were within the optimal range only when extraction was performed using the DNeasy method with *Scophthalmus maximus* and *Sepia officinalis* samples [22].

#### 3.1.3. Integrity of Extracted DNA

Another important variable that can influence the PCR performance of a DNA extract is integrity, defined as the level of degradation of the DNA extract, i.e., a degradation process in which DNA is broken down by biological (nucleases activity), chemical and physical processes. Knowing whether the DNA has been degraded with processing is important when designing downstream applications; therefore, analysis of the extracted DNA is crucial. DNA integrity was evaluated in this study by agarose gel electrophoresis of DNA extracts; however, only the Wizard and MPure-12 methods provided enough DNA to allowing this analysis. In the Supplementary Materials, Figures S1 and S2 present the electrophoresis of DNA samples extracted with these two methods. The results were similar in terms of the range of fragment sizes obtained: between 9000 and 24,000 bp; this result guarantees that PCR amplification would not be limited by the integrity of the DNA present in the extract [8]. In the gel, it was possible to observe some differences regarding the species analyzed; in the case of hake (*M. merluccius*) and cod (*G. morhua*), a band with a value higher than 9416 bp was clearly distinguished. However, this band was not detected in the case of the other species analyzed.

### 3.2. Handling Time and Total Extraction Time

The total DNA extraction time varied considerably depending on the methodology used (Table 1). Chelex and swab extraction were very fast compared to the other methods.

### 3.3. PCR and Sequencing

**Figure 7:**
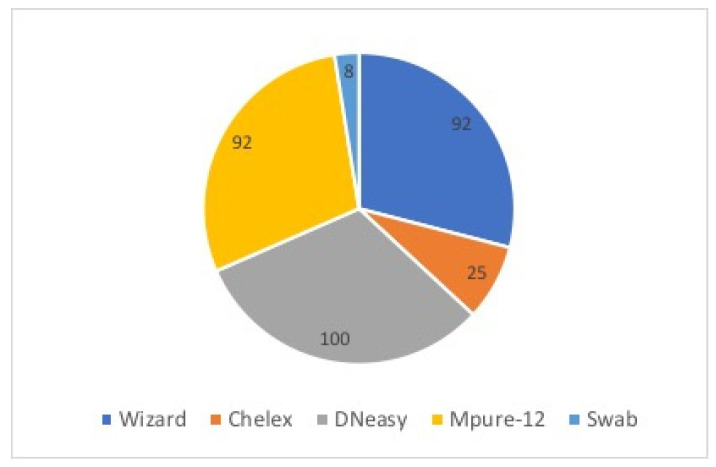
Total amplification success (%) of the tested samples per DNA extraction method.

With respect to PCR products, DNeasy was the best method in terms of amplificability, achieving 100% amplification success (Figure 7). In addition, the amplification success of the Wizard and MPure-12 methods was high with 11 amplifications. The poorest amplification success was obtained with the swab method, with only one sample showing a clear band (Table 2). The electrophoresis gels of these results are presented in Supplementary material (Figures S3 and S4).

The quality of the obtained DNA sequence was evaluated and expressed in percentage (Table 3). Because the fragments were different sizes, we were not able to compare the sequence quality; however, we did compare the quality obtained among methods within each species group. The highest percentage of sequence quality was obtained with the MPure-12 DNA extraction protocol for both Burgener and Folmer primers. The lowest percentages were obtained with Chelex and DNeasy methods for both fragments of primers. These results demonstrate the relation between the ng of DNA loaded per reaction and the quality of sequencing. More ng of DNA implies better quality. In fact, the amount of DNA that could be loaded for the Chelex method was so small that only one sample out of three was successfully sequenced. Figure S5, shows a sequence view (quality of 100%) of the *Merluccius merluccius* sample obtained with the Wizard methodology.

Based on the species authentication, phylogenetic trees were created using the neighbor-joining method with the Tamura–Nei model with 1000 bootstrap replicates [21] (Figures S6 and S7). The Blast results for the authentication of the individuals are shown in Table 4.

### 3.4. Complementary Parameters

After analyzing and comparing the methods according to their characteristics (Table 5 and Table 6), we concluded that the safest methods are the Chelex and swab protocols, as the use of these methodologies did not involve hazardous components or dangerous substances. Conversely, the most dangerous method in terms of hazardous components classification is the DNeasy extraction kit. The most affordable and most expensive methods, by far, compared to the other protocols in terms of reagent cost are the Chelex and DNeasy methods, respectively. Nevertheless, the DNeasy method did not require any specific equipment, which was not the case for the MPure-12 method. The simplest working procedure to carry out is the swab methodology, although MPure-12 has the possibility of automation, contrary to the other methods.

## 4. Discussion

The results evidence significant differences in terms of yield, efficiency and purity obtained from different DNA extraction methods (*p* < 0.05), (*n* = 60).

### 4.1. DNA Quantity and Quality

The differences in DNA yields observed with the tested DNA extraction methods were very large in favor of the Wizard method. The lowest yields were observed in the case of Chelex and swab methods. Total DNA extracted (yield) is a very important aspect to determine the suitability of a method for a particular application; nevertheless, when analyzing the yield values, the higher amount of initial material used in the Wizard method compared to the other methods must be taken into account. Some of the methods evaluated involved the use of proteinase K, such as the Wizard, DNeasy and M-pure methods. However, the amount used in the two latter methods is lower than in the case of the Wizard method (400 and 800 microg, respectively); thus, the highest yield obtained with the Wizard method might also be explained by a higher DNA release from proteins [6,12]. Determining the efficiency (ng of extracted DNA per mg of tissue) of each method allows for comparison between protocols.

The method with the highest efficiency was the Chelex protocol, in spite of the low yield achieved; results were similar to those reported by Besbes et al. fresh, sardine anchovy [10] and tuna [23]. This efficiency might be explained by the simple procedure and the reduction in DNA degradation during the process [9]. Singer-Sam et al. postulated that the presence of Chelex during boiling prevents the degradation of DNA by chelating metal ions that may act as catalysts in the breakdown of DNA at high temperatures in low-ionic-strength solutions [24].

Obtaining high DNA concentration and yield is an advantage of providing templates for PCR-based applications. However, consistency and reproducibility are also important factors in any analytical procedure; in the case of a DNA extraction, purity is also essential achieve successful amplification. Furthermore, we observed that DNA concentration in the extract also favors the amplification of highly degraded DNA, as it is the case with canned samples [7]. Furthermore, the method to be chosen will depend on the ultimate purpose of the analytical procedure, the type of sample, the level of processing and the number of samples to be analyzed [12].

Although the characteristics of the starting material (in this case, muscle tissue) may vary depending on the species (i.e., cephalopod compared with fish), there were not notable differences in the DNA extraction when the same DNA isolation method was used. If we compare the data among Pleuronectiformes, Gadiformes and Cephalopoda, no significant variability between species was observed.

We used A260/280 as a primary measure of purity in DNA extracts. The optimal A260/280 for DNA extracts is 1.8, whereas values of 2.0 may be used for the assessment of RNA extracts. Values lower than 1.8 may indicate low amounts of nucleic acids or the presence of proteins and/or extraction contaminants, such as phenol. In terms of purity, the methods that are based on binding nucleic acids to a matrix, such as the Wizard, DNeasy and MPure-12 methods, exhibited the best DNA quality, in accordance with the results obtained in previous studies [7,12]. Other methods achieved results very close to the optimal range in terms of the A260/A280 ratio but with a high standard deviation among individuals. This is the case of the Chelex technique, which achieved better DNA quality values in this study (A260/A280 = 1.74) than in those reported by Besbes (A260/A280 = 1.6–1.5) [10], but with a significant standard deviation (±0.28). The presence of RNA in a sample may increase the A260/A280 ratio due to both DNA and RNA absorbing ultraviolet light at a wavelength of 260 nm. As a consequence, spectrophotometry is not able to distinguish between DNA and RNA [12]. Moreover, low values of the A260/A280 ratio could indicate the presence of residual agents or proteins, which could explain the poor results obtained with the swab method. In order to detect possible carbohydrate contamination, the ratio A260/A230 was tested [22]. In this study, the data ratio ranged from 0.44 ± 0.15 to 2.36 ± 0.11. Only the DNeasy and MPure-12 methods were obtained near-optimal value (2.00–2.20), indicating a low concentration of contaminants. The A260/A230 values associated with the tother methodologies were far from the optimal range, which could be due to traces of aromatic substances [10]. The presence of contaminants evidences the low capacity of the Chelex and swab methods to eliminate impurities that may inhibit PCR [3].

Besides obtaining DNA of good quality, the aim of an isolation protocol is to reduce the quantity of degraded DNA [3]. The integrity of the extracts was used as an additional measure to understand the success rate of PCR amplifications. The DNA integrity was very similar for both the Wizard and the MPure-12 methods, representing the most successful methods in terms of PCR amplification, together with DNeasy. All fragments were amplified without signs of degradation or contamination. Samples of *Sepia officinalis* show bands of high molecular weight. This species is probably captured by inshore fishing in the Ría de Vigo, reaching the fishmonger in a short time and preserving the freshness of the product with its tissue practically intact. Nevertheless, samples of *Gadus morhua* also present a high-molecular-weight band of 9416 bp. This result was not expected, as cod is usually a species captured in deep-sea fishing in areas far from Galicia, which may result in some tissue degradation because of the time elapsed since the capture moment. Tissue degradation by endogenous enzymes takes place after the death of the fish. The extent of autolysis of the tissue depends on the time the fish is on ice before DNA extraction, as well as the sensitivity of mitochondria to freezing and/or the level of nuclease activity.

### 4.2. Handling Time and Total Extraction Time

The Chelex and the swab methods were the fastest DNA extraction protocols. On the contrary, the Wizard and the DNeasy methods were the methods that required the most steps and human handling. However, simplification and reduction in handling may involve also decrease some DNA extract features, such as purity, as we have found in this study. Thus, the speed offered by the very simple Chelex and swab methods entails a reduction in quantity and quality of the obtained DNA obtained. However, when processing a large number of samples, protocols that require fewer steps and less time may be more adequate [9]. For some downstream applications, it may be worth investing more global time in extraction in order to obtain DNA of better quality, although more total extraction time does not necessarily result in purer DNA. There is the special case of the MPure-12 method, which involves three hours of total DNA extraction time, including one hour of non-attended time, with superior results in terms of purity. This method may replace the manual work of laboratory personnel during the process, and this time saved could be reflected in economic benefits [3].

### 4.3. PCR and Sequencing

The DNA extraction quality was also assessed for its amplificability. The DNA damage induced by the isolation process, the length of DNA fragments and the presence of inhibitors influence the success of amplification [2,10,23]. Nevertheless, PCR is an efficient technique owing to its ability to work with poor-quality DNA [9].

As other authors have shown, the DNeasy, MPure-12 and Wizard can be considered the best among the tested methods because of the total amplification success rate accomplished [7,13]. The percentage of amplification success achieved by Helge et al. [25] with the DNeasy method for otoliths, scales, fins and gill tissue from European whitefish was 100%, as in this experiment for muscle tissue, which implies a lower percentage of PCR repetitions and sequencing and therefore savings in cost and time. MPure-12 and Wizard methods successfully amplified 11 of the 12 samples. The good performance of these methods can be attributed to the integrity and purity of the obtained DNA fragments and the low level of DNA damage during extraction [10]. Conversely, amplicons were not obtained when using the Chelex and swab methods, especially for Pleuronectiformes. The initial low DNA quantity achieved with these methods could be insufficient for the primers to bind to the template, leading to DNA not being amplified and detected in the gel [3,9,10,12]. Not only a low quantity of DNA but also a high quantity in the PCR could be associated with an excess of inhibitors [6]. In addition, with the Chelex and swab methods no sample digestion or purification step is carried out.

The most suitable technique for identifying species based on information obtained from the PCR products is DNA sequencing [6]. The MPure-12 and Wizard methods achieved the best sequence qualities for both fish (99.2 ± 0.2, MPure-12; 99 ± 0.5, Wizard) and cephalopods samples (94.3 ± 1.3, MPure-12; 93.2 ± 1.1 Wizard), showing similar data to those of other works [13]. Of all the PCR products obtained with all tested methods, only three belonging to *Gadus morhua* samples failed in the sequencing runs. Two of these sequencing failures belong to the same swab sample and the third to a DNeasy sample that produced only one strand. Additionally, a *Gadus morhua* sample did not generate a clean sequence, so it was considered unsuccessful. There is no apparent relation between the purity or the concentration of the extracted DNA and the sequencing failures. Nonetheless, poor sequencing results may occur due to the presence of impurities in the sample [13], which could explain the swab samples failures.

## 5. Conclusions

The ideal methodology for DNA extraction depends on the tissue and the ultimate application. If the priority is to quickly and easily extract DNA regardless of the quality, the Chelex and swab methods are the best options. Besides being the cheapest, these are safest protocols and do not involve the use of toxic materials or specific equipment, providing sufficient quality for the amplification of small fragments in some samples. In spite of the higher cost of reagents, if we are interested in obtaining a better-quality DNA extract (e.g., for the amplification of long fragments), the Wizard, DNeasy and MPure-12 methods are the most suitable. The MPure-12 method has the possibility of automation, which can be initially expensive but very useful when handling a very large number of samples. The selection of the extraction methodology will depend on our capabilities and work needs.

## Figures and Tables

**Table 1:** Digestion time, handling time and total extraction time of the tested methods.

Method	Digestion Time	Handling Time	Total Extraction Time
Wizard	2 h	3 h	5 h *
Chelex	-	1 h 30 min	1 h 30 min
DNeasy	2 h	2 h	4 h
MPure	2 h	1 h	3 h *
Swab	-	1 h	1 h

* Wizard and MPure methods are standardized to 10 and 12 samples, respectively.

**Table 2:** Amplification success of PCR products of the tested species per method.

Sample	Wizard	Chelex	DNeasy	MPure	Swab
SMAX	*+ + +*	− − −	*+ + +*	*+ + +*	− − −
MMER	*+* − *+*	− *+* −	*+ + +*	*+* − *+*	− − −
GMOR	*+ + +*	− − −	*+ + +*	*+ + +*	*+* − −
SOFF	*+ + +*	*+ +* −	*+ + +*	*+ + +*	− − −

**Table 3:** Sequence quality percentage of the tested DNA extraction methods. Data are presented as Mean ± standard deviations of samples per method.

Method	Total DNA Loaded (ng) per PCR Reaction	Sequence Quality % Burgener Primers	Sequence Quality % Folmer Primers
Wizard	100	99 ± 0.5	93.2 ± 1.1
Chelex	11.5 ± 5.7	89.2	-
DNeasy	26.1 ± 19.1	97.3 ± 4.6	84 ± 15
MPure	100	99.2 ± 0.2	94.3 ± 1.3
Swab	9 ± 3.05	-	-

**Table 4:** Blast results for the authentication of the samples tested in this study.

Sample	GenBank Accession Number	Species	Query Cover (%)	% of Identity	Date of Access
SOFF1	ON564881	*Sepia officinalis*	100	100	11/05/2022
SOFF2	ON564882	*Sepia officinalis*	100	100	11/05/2022
SOFF3	ON564883	*Sepia officinalis*	100	100	11/05/2022
GMOR1	ON505202	*Gadus morhua*	100	100	11/05/2022
GMOR2	ON505203	*Gadus morhua*	100	100	11/05/2022
GMOR3	ON505204	*Gadus morhua*	100	100	11/05/2022
MMER1	ON505205	*Merluccius merluccius*	100	100	11/05/2022
MMER2	ON505206	*Merluccius merluccius*	100	99.76	11/05/2022
MMER3	ON505207	*Merluccius merluccius*	100	100	11/05/2022
SMAX1	ON505208	*Scophthalmus maximus*	100	100	11/05/2022
SMAX2	ON505209	*Scophthalmus maximus*	100	100	11/05/2022
SMAX3	ON505210	*Scophthalmus maximus*	100	100	11/05/2022

**Table 5:** Hazardous components and substance classification of the DNA extraction kits.

	Wizard	Chelex	Dneasy	Mpure-12	Swab
Hazardous components and substance classification	Proteinase k solution (GHS08) Guanidinium thiocyanate (GHS05, GHS07) Isopropanol	-	Proteinase k solution (GHS08) Guanidinium chloride (H302 + H332, H315, H319), maleic acid (H302, H312, H315, H319, H317, H335)	Proteinase k solution (GHS08) Guanidinium chloride (H302 + H332, H315, H319)	-

**Table 6:** Parameters measured for comparison of the tested DNA extraction methods. In the case of non-quantitative variables, the measurement scale ranges from + to ++++ (minimum to maximum, respectively). The measurements are highlighted according to a color scale (green, yellow, orange, and red), indicating a scale of values, from best (green) to worst (red).

	Wizard	Chelex	DNeasy	MPure-12	Swab
Yield (Total DNA (ng))	11,404.72 ± 4307.16	194.66 ± 111.16	787.64 ± 721.96	2653.06 ± 1372.63	892.92 ± 336.08
Efficiency (ng DNA/mg wet tissue)	38.108 ± 14.943	68.393 ± 43.454	36.523 ± 33.292	66.843 ± 36.349	17.505 ± 7.098
Purity (260/280)	1.967 ± 0.054	1.843 ± 0.526	2.177 ± 0.263	2.031 ± 0.184	1.547 ± 0.159
Rapidity (Extraction time)	5 h *	1 h 30′	4 h	3 h *	1 h
PCR amplification success %	94.44	33.33	100	99.44	5.55
Safety of components (see Table 4)	+ +	+ + ++	+	+ + +	+ + + +
Affordability (reagent cost per prep)	EUR 2.22	EUR 0.001918	EUR 4.26	EUR 5.65	EUR 0.051
Affordability (specific equipment value)	Vacuum manifold	Not required	Not required	MPure-12^TM^ automated nucleic acid purification system	Not required
Technical simplicity	+	+ + +	+ +	+ +	+ + + +
Automation	-	-	-	+ + + +	-

* Wizard and MPure methods are standardized to 10 and 12 samples, respectively. The measurements are highlighted according to a color scale (green, yellow, orange, and red), indicating a scale of values, from best (green) to worst (red).

## Data Availability

All related data and methods are presented in this paper. Additional inquiries should be addressed to the corresponding author.

## Supplementary Materials

The following supporting information can be downloaded at: https://www.mdpi.com/article/10.3390/foods11121739/s1, Figure S1: 1% agarose electrophoresis gel of Wizard extracts. One specimen per species; Figure S2: 1% agarose electrophoresis gel of MPure-12 extracts. One specimen per species; Figure S3: PCR products for cythocrome b of (a) Wizard method; (b) MPure-12 method; (c) Chelex method; (d) DNeasy method and (e) Swab method on 2% agarose electrophoresis gel. Scophthalmus maximus (SMAX), Merluccius merluccius (MMER) and Gadus morhua (GMOR); Figure S4: PCR products for cythocrome c oxidase I (COI) of (a) Wizard method; (b) MPure-12 method; (c) Chelex method; (d) DNeasy method and (e) Swab method on 2% agarose electropho-resis gel. Sepia officinalis (SOFF); Figure S5: Sequence view of Merluccius merluccius from the Wizard method (100% sequence quali-ty); Figure S6: (a) Neighbor-Joining phylogenetic trees for cythocrome b of pleuronectiformes; (b) Neighbor-Joining phylogenetic trees for cythocrome c oxidase I (COI) of Cephalopoda; Figure S7: Neighbor-Joining phylogenetic trees for cythocrome b of Gadiformes.
