# Supplementary material for: The Influence of DNA Extraction Methods on Species Identification Results of Seafood Products
Source: Foods. 2022 Jun 14;11(12):1739. doi: 10.3390/foods11121739 (PMC9222224; doi:10.3390/foods11121739)
Supplement: Supplementary file 1 [file foods-11-01739-s001.zip › foods-1703310-supplementary.pdf]

Supplementary materials

The influence of DNA extraction methods on species identification results of seafood products

Rosalía Rodríguez-Riveiro [1], *, Amaya Velasco [1] and Carmen G. Sotelo [1]

[1] Instituto de Investigaciones Marinas (CSIC), Eduardo Cabello 6, 36208 Vigo (Spain); amaya-velasco@iim.csic.es ,carmen@iim.csic.es
* Correspondence: rosalia@iim.csic.es

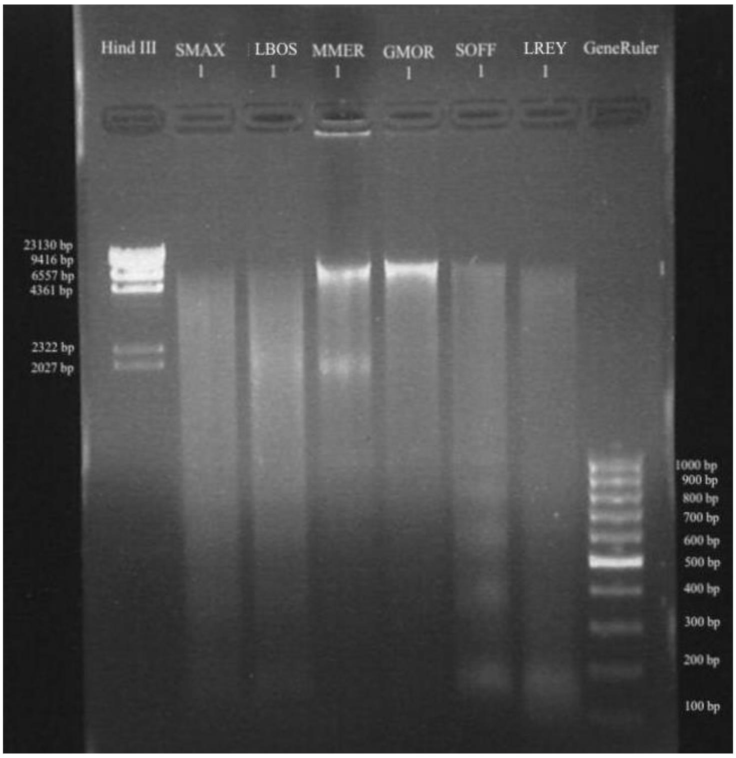

Figure S1. 1% agarose electrophoresis gel of Wizard extracts. One specimen per species.

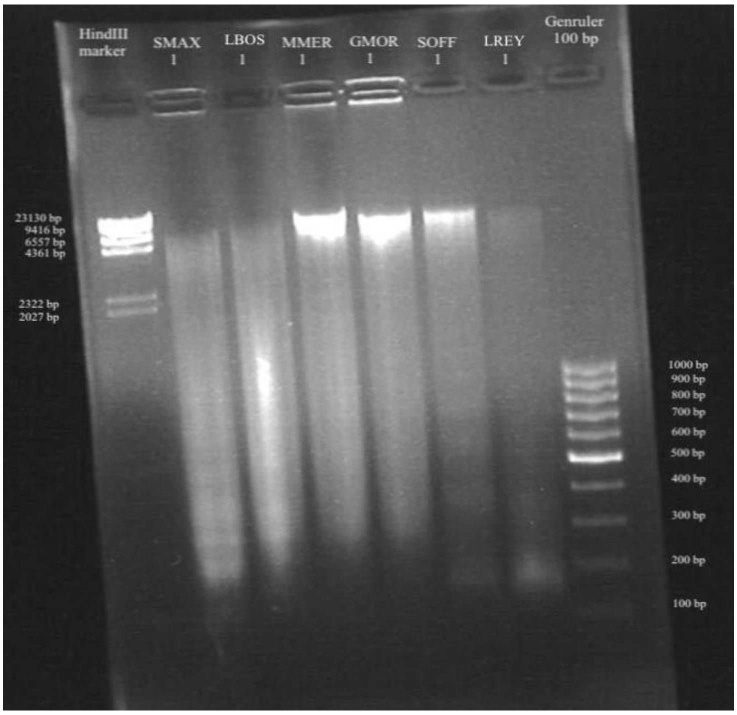

Figure S2. 1% agarose electrophoresis gel of MPure-12 extracts. One specimen per species.

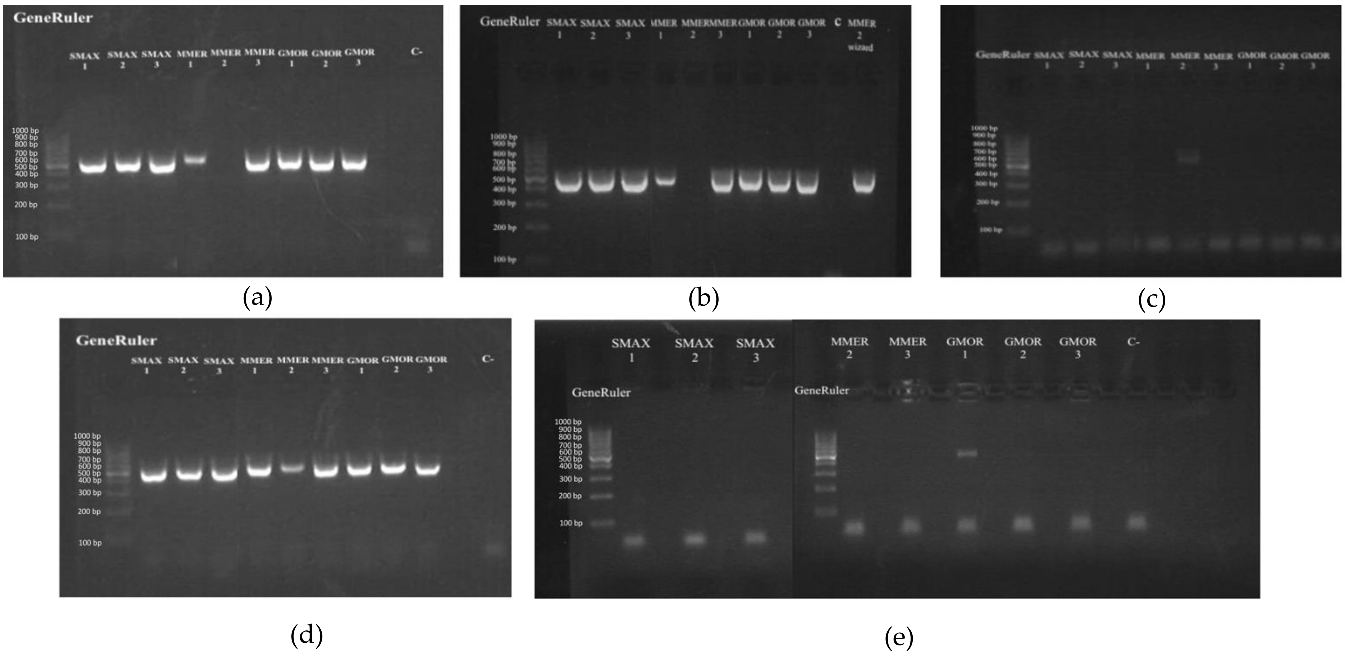

Figure S3. PCR products for cythocrome *b* of (a) Wizard method; (b) MPure-12 method; (c) Chelex method; (d) DNeasy method and (e) Swab method on 2% agarose electrophoresis gel. *Scophthalmus maximus* (SMAX), *Merluccius merluccius* (MMER) and *Gadus morhua* (GMOR).

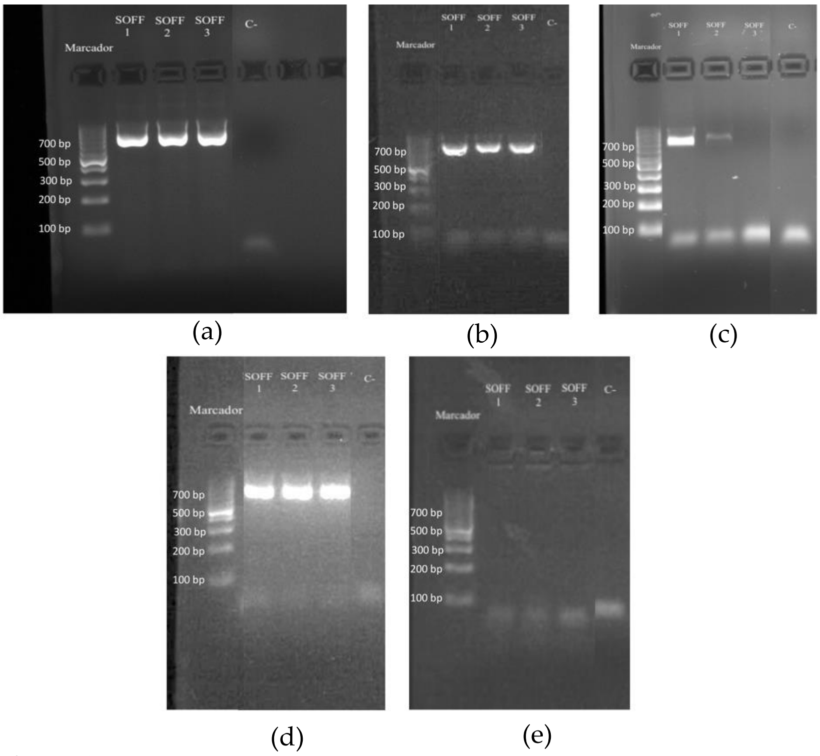

Figure S4. PCR products for cythocrome *c* oxidase I (COI) of (a) Wizard method; (b) MPure-12 method; (c) Chelex method; (d) DNeasy method and (e) Swab method on 2% agarose electrophoresis gel. *Sepia officinalis* (SOFF).

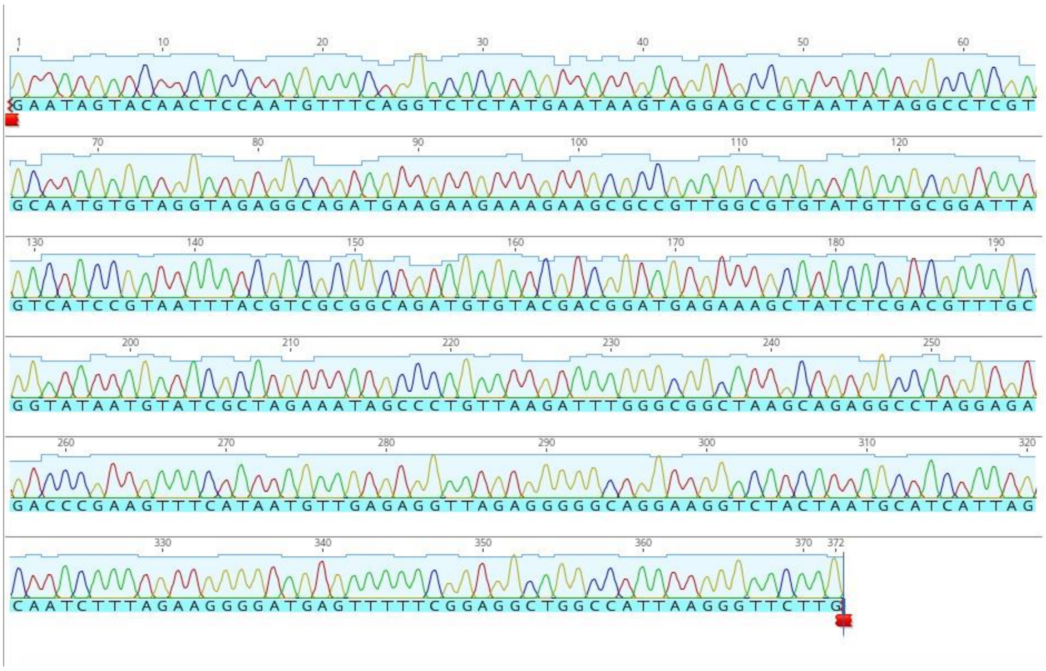

Figure S5. Sequence view of *Merluccius merluccius* from the Wizard method (100% sequence quality).

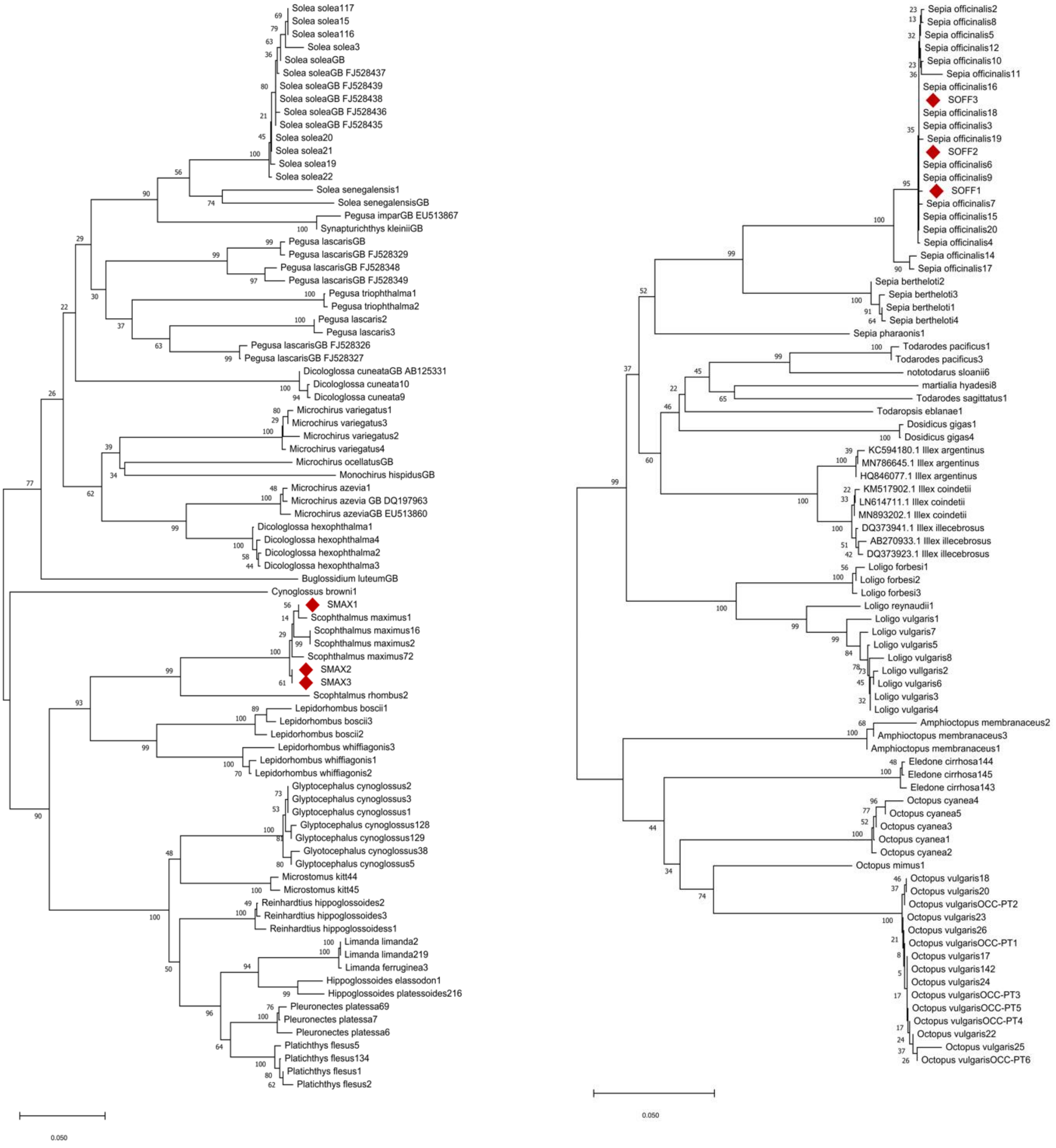

(a) (b)

Figure S6. (a) Neighbor-Joining phylogenetic trees for cytochrome *b* of pleuronectiformes ; (b) Neighbor-Joining phylogenetic trees for cytochrome *c* oxidase I (COI) of Cephalopoda.

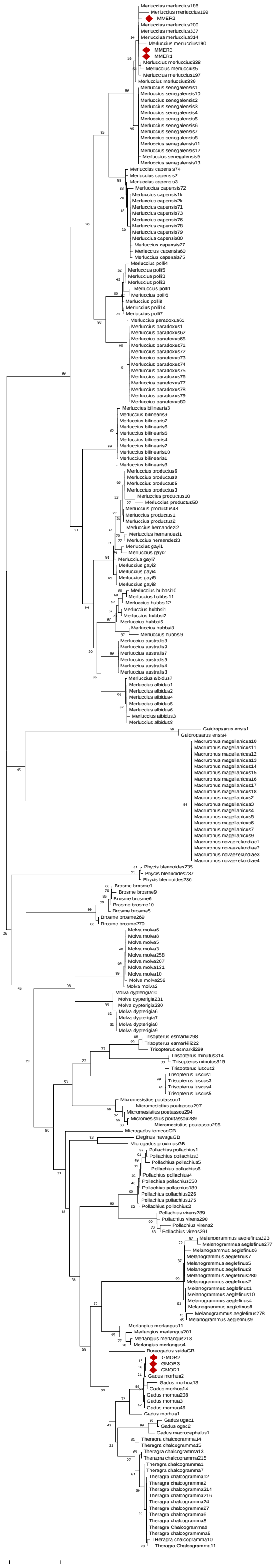

Figure S7. Neighbor-Joining phylogenetic trees for cythocrome *b* of gadiformes.
